# Anomie, irritation, and happiness in the Chilean society post-social outbreak

**DOI:** 10.3389/fpsyg.2023.1145121

**Published:** 2023-04-11

**Authors:** Emilio Moyano-Díaz, Gonzalo Palomo-Vélez, Pablo Olivos-Jara, Yuxa Maya-López

**Affiliations:** ^1^University of Talca, Talca, Chile; ^2^Universidad de O’Higgins, Rancagua, Chile; ^3^Universidad Castilla la Mancha, Albacete, Spain

**Keywords:** anomie, social outbreak, political irritation, happiness, well-being, mediation

## Abstract

On 18 October 2019, the Chilean people witnessed an unprecedented social outbreak across most of their country. We argue that a state of anomie is a factor associated with the weakening of states, and an anomic state might negatively influence people’s well-being through an increased feeling of irritation. Convenience recruitment *via* social networks allowed us to form a sample of 194 Chilean participants from the center-south region of the country (*M* = 36.53 years old, SD = 17.48; 56.7% women). All participants completed testing instruments to measure anomie, irritation, happiness, and political beliefs. Descriptive scores suggest situating Chile in the quadrant of high anomie. Two mediation analyses were conducted. The main results showed a negative indirect effect of the breakdown of the social fabric and leadership on happiness through irritation, although the findings for the former dimension were more robust. Additionally, the breakdown of the social fabric was positively related to the belief that left and right-wing democratic governments are helpless when it comes to fighting delinquency. The breakdown of leadership, on the other hand, was negatively related to political interest. The results should be interpreted with caution due to the limitations of the sample type and the construction validity of some instruments.

## 1. Introduction

On 18 October 2019, the Chilean people witnessed an unprecedented social outbreak across most of their country. Thousands took to the streets to demand social changes—with the biggest public demonstration bringing together more than 1.2 million people just a few days later in Chile’s capital city, Santiago (BBC News Mundo, 2019). Indeed, the outbreak was of such magnitude that it even threatened the continuity of the right-wing government of the time. The tipping point was represented by a somewhat minor incident—as is typically the case (Paez and Da Costa, 2022), namely, a 30-peso increase (i.e., 0.03 USD) in Santiago’s public transport system fares. Although the citizen demands during the outbreak did not insist on profound and/or structural changes to the dominant model—as was seen, for example, during the education-related Chilean social outbreak of 2011 (Mira, 2011), they did call for improvements in the quality of education for all, increases in pensions, better health care, and, especially, a halt to abuse and undignified treatment. However, the political class channeled these demands by agreeing to a change to the current Constitution, the origin of which lies in the dictatorship of Pinochet (enacted in 1980), through a democratically elected constituent convention of 154 members.

This agreement for a new political constitution and the advent of the COVID-19 pandemic in March 2020 led to an abatement of the frequent demonstrations and street disorders to the point of just a few isolated cases. That said, in September 2022, the new proposal for the constitution was overwhelmingly rejected in a national plebiscite, obtaining only 38% approval. The country is currently immersed in the process of building a new constitution, in an uncertain and socially divided environment.

In the current research, we argue that a state of anomie, understood as a generalized perception of normlessness—mostly derived from the perceived lack of effective leadership and the erosion of moral standards (Teymoori et al., 2016, 2017), is a factor associated with the weakening of states and which affects people’s well-being. First, theoretical considerations of anomie and the relationship of the phenomenon to the social outbreak are described. We then delve into how an anomic state might negatively influence people’s well-being through an increased feeling of irritation.

### 1.1. Societal and individual factors behind the Chilean social outbreak

It has been suggested that different factors underlie the social outbreak, including a long-standing anomic process (Moyano-Díaz et al., 2021), and the dominant neoliberal socioeconomic model and its consequences (i.e., neoliberalism; Garretón, 2021). These include inequalities, and, particularly, the irritation of those who feel marginalized and exploited (Castro-Abril et al., 2021) and whose dignity has been wounded. Different explanations have been proposed—although most are ideological or political rather than empirical—regarding the reasons that led to the social outbreak of 2019.

Morales (2020), for instance, suggests that a social outbreak of these characteristics was foreseeable, as the country was in the grip of four significant crises: (1) a crisis of participation—exacerbated by voluntary voting since 2012, (2) a crisis of representation, that is, lower adherence to political parties and loss of confidence in key democratic institutions (e.g., government, courts, and congress), (3) a crisis of trust in public institutions and those related to social order (e.g., police, the church), and finally, (4) a crisis of probity, with frequent cases of irregular funding of politics and business collusions. In a similar vein, a recent study also points to the lack of trust and confidence in municipal political institutions and a general misidentification with the elite and the government, as factors leading to the outbreak (Castro-Abril et al., 2021). Particularly, analyzes of nationally representative survey data on Chilean adults from 2015 to 2019 suggested that perceived corruption in key institutions (e.g., parliament, army, police, municipalities, churches, congress, courts of justice, political parties, and even in sports organizations) significantly increased during the 6-year period observed (Moyano-Díaz et al., 2021). This lack of trust in institutions, however, does not seem to be recent (Segovia et al., 2008; Mira, 2011) or to be seen only in Chile. Indeed, the Chilean social outbreak is not an isolated case at global level. Social outbreaks of similar characteristics have been reported in Africa (e.g., Blanes, 2017), Asia (e.g., Lytkina, 2015), the Middle East (e.g., Leila et al., 2010), and other Latin-American countries (Amador-Baquiro and Muñoz-González, 2020). More importantly, however, massive social demonstrations seen in countries such as Haiti, Honduras, Peru, Venezuela, Colombia, Ecuador, Chile, and Bolivia seem to respond to specific problems and needs felt by the public, with a common factor being widespread discontent with those who govern (Marín and Mesa, 2019). Indeed, in a qualitative sociological interpretation of the Chilean social outbreak, irritation is proposed as the trigger. The process is considered to begin with excesses in societal demands, of inequalities in interactions, or in the use of power—all characteristics closely related to the emergence of anomic states, followed by disenchantment due to unfulfilled social promises, leading, in turn, to irritation and anger. Irritation is also seen as being linked to inequalities, heightened awareness of abuse, and lack of courtesy and civility, and aggressiveness in daily urban life (Araujo, 2019).

Despite the emergence and topicality of the subject, in Chile, the social sciences in general, and social psychology in particular, have shown very little interest in the study of anomie. One exception is a study comparing the levels of anomie reported by a sample of high school students during the Chilean dictatorship in 1989, and those reported by a relatively comparable sample of high school students in democracy, in 2007 (Aceituno et al., 2009). The results suggested that, despite democracy having been recovered, anomie levels among the students did not differ from those seen in a period of great social instability. Furthermore, Aceituno et al. (2009) understood anomie as a state in which society stops exercising a regulatory role over the passions and aspirations of individuals, diminishing its ability to impose limits on what people can want or do. Thus, the sense of social cohesion is weakened, where the rules, norms and social purposes are not valid in daily life. This could well characterize Chilean society today.

### 1.2. Anomie and well-being

From its origins, the concept anomie was described as the effects on groups and individuals of the absence of norms in institutionalized social structures (Durkheim, 1933; Merton and Merton, 1968). It is a shared social perception at the macro-social level and whose causes may lie in political, economic, or social realities, such as rapid growth, structural crises, economic inequality, war, or severe social conflicts. Currently, in addition to the classic economic factors, such as inequality, other factors, such as the crisis of political representation and others already mentioned, an economic crisis (inflation and low growth) exacerbated by the pandemic, the globalized economic effects of a war (Ukraine-Russia), together with a dramatic increase in homicides, have incorporated into anomie a measure of social disorganization and, above all, fear and insecurity.

The Chilean economy is suffering its worst inflation in 30 years (Banco Central, 2022), and will be, along with Brazil, one of the lowest growing Latin-American economies in 2023 (World Bank, 2022). Feelings of insecurity and fear due to uncontrolled crime—particularly in the specific form of homicides, are common (Ajzenman et al., 2021).

In addition, the inadequate leadership of the (right-wing) government authorities shortly before the social outbreak may also have contributed to an anomic process, with ministers (especially those of health and the economy), making statements in the media that could easily be interpreted as irony or mockery of the common citizen, demonstrating an extraordinary distance and lack of empathy with the population (Moyano-Díaz et al., 2021).

Conceptually, for Teymoori et al. (2017), anomie has two dimensions: perception of a break or fracture in the “social fabric,” that is, disintegration as a lack of trust and moral standards, and a perceived break or collapse in leadership (deregulation as lack of legitimacy and leadership effectiveness). Building on Haslam et al. (2020) and on Reicher et al. (2005), and Teymoori et al. (2017) propose that these two dimensions are orthogonal and tend to reinforce each other. An effective and legitimate leadership promotes and bolsters the social fabric, and if this social fabric is strong—that is, cohesive and unified—people are likely to choose leaders that are more prototypical or representative of the collective. The possibilities are then four: (1) when both dimensions are perceived as having little or no fracture, anomie is low; (2) when leadership is perceived as highly fractured but the social fabric is perceived as healthy, there is disintegration; (3) when leadership is perceived as healthy but the social fabric is highly fractured, there is deregulation; and finally (4) when people perceive both the social fabric and leadership as highly fractured, then society is experiencing anomie.

Teymoori et al. (2016) argue that anomie emerges when both a breakdown in leadership and a breakdown in the social fabric co-occur, and that anomie primarily undermines well-being and life satisfaction.

To measure anomie, Teymoori et al. (2016) proposed their perception of anomie scale (PAS), which was applied in 28 countries, including Chile (151 students), before the social outbreak of 18 October. The results showed that the PAS correlates positively with national indicators of social functioning and predicts national identification and well-being. In the case of the sample of Chilean students, the participants reported a moderately high level of anomie with an average score of 4.53 (out of 7) on the global scale and similar rates in both its core dimensions (i.e., 4.47 for the breakdown of the social fabric and 4.6 for the breakdown of leadership). Furthermore, the authors suggest that countries with less strong or developing economies, such as Chile, tended to score higher on anomie, while others, from Northern Europe, with higher GDP and development, tended to show less anomie (i.e., averages lower than 4; Teymoori et al., 2016). Indeed, such results are aligned with those of Aceituno et al. (2009), who reported relatively high levels of anomie among Chilean students in both 1989 and 2007, and with those of Muratori et al. (2013) in Argentina. The authors reported high levels of anomie, suggesting individuals’ difficulties in perceiving themselves as engaged with their environment, that is, feelings of distance from leaders, perceived disorganization of society, and the impossibility of meeting individual and trivial goals. Moreover, authors have also suggested that trust in institutions (Inglehart et al., 2004) is negatively associated to perceptions of anomie. Finally, a Mexican study has shown negative associations between perceptions of anomie and social well-being (e.g., the feeling that one is an important part of the community), rejection of democratic institutions, and interest in politics (Laca Arocena et al., 2011). Regarding this last variable, we hope to find a relationship in the same negative direction in the present study. Inspired by Teymoori et al. (2016), it is expected to find an association between anomie and political hopelessness regarding the fight against crime.

### 1.3. The path from anomie to well-being: The role of irritation

The construct “irritation” was first introduced in Germany by Mohr (1986), who described it as dealing with the experience of uncertainty and corresponding reactions. In psychological terms, uncertainty may arise when an individual experiences a discrepancy between a given situation and an important personal goal. Irritation can be viewed as a deteriorating state of mind, resulting from a perceived mismatch of goals (Mohr et al., 2006). It is of great interest to test the hypothesis of irritation as a possible component of the malaise attributed to Chilean society, and its relationship to anomie and, particularly to happiness, a virtually unexplored variable in this regard.

It is also unknown, if the existence of anomie were confirmed, in which quadrant Chilean society would be located. Determining this could help explain the social turbulence present in Chile, because, on the one hand, ideological, rather than scientific, explanations predominate and, on the other, the local social sciences community has failed to delve deeply into the empirical study of these dimensions. Nor is it known how anomie is related to irritation and happiness, although a positive relationship may be expected with the former and a negative relationship with the latter. Thus, our purpose is twofold: (i) to determine whether there is a perception of anomie, in its two dimensions, in Chilean adults and how much, and (ii) to identify the eventual mediating role of irritation in the effects of anomie on happiness.

## 2. Methods

### 2.1. Participants

A convenience sampling method was used to recruit participants *via* social networks. The sample comprised 194 Chilean participants from the center-south region of the country, whose ages varied between 18 to 81 years old (*M* = 36.53, SD = 17.48). Of these participants, 56.7% were women. Regarding education, 26.3% had primary/secondary studies, 13.9% had vocational training, and 59.8% had university studies.

### 2.2. Instruments

An online questionnaire was developed using the Google Docs software. Three instruments measuring anomie, irritation, and happiness were used. Furthermore, the participants also responded to single items measuring political beliefs and socio-demographic data (sex, age).

Anomie: To measure anomie, a Spanish version of the Perception of Anomie Scale was used (PAS; Teymoori et al., 2016). The PAS consists of 12 seven-point Likert-type items (1 = totally disagree to 7 = totally agree) that measure general anomie and its two core dimensions; the breakdown of the social fabric (e.g., “People think there are no clear moral standards to follow,” “Everyone thinks of him/herself and does not help others in need”) and the breakdown of leadership (e.g., “Some laws are not fair,” “Politicians do not care about the problems of the average person”). The PAS has shown adequate reliability for each of its dimensions as well as for the overall scale (Teymoori et al., 2016). In the current study, the PAS also showed an adequate reliability for each dimension (α social fabric = 0.68; α leadership = 0.74) and for the complete scale (α social fabric = 0.74). Exploratory Factor Analysis (principal axes, oblique rotation) yielded three factors that explain 53.96% of the variance. Eigenvalues and content analysis of their items suggest a reduction to the two factors expected, and new studies to improve two items of the breakdown of leadership in the future.

Irritation: To measure irritation, an adapted Spanish version of the Irritation Scale was used (Mohr et al., 2006). The Irritation Scale consists of 8 seven-point Likert-type items (1 = totally disagree to 7 = totally agree) that measure irritation in occupational contexts. For this study, we adapted the scale to measure irritation with the Chilean political context. For instance, the item “Even on my vacations, I think about my problems at work” was modified to “Even on my vacations, I think about the political problems of my country or city,” the item “I anger quickly” was modified to “I anger quickly, especially when I hear politicians talk” (for the complete adapted scale, see Annex 1). The Irritation scale has shown adequate reliability in past literature (Mohr et al., 2006) and our adapted version also showed adequate reliability (*α* = 0.79). An Exploratory Factor Analysis forcing the extraction of a factor (principal components) yielded an explained variance of 42% with eigenvalues greater than.400.

Annex 1.- Irritation scale (Mohr et al., 2006) adapted to the political context.

1. Tengo dificultad para relajarme después de ver o leer entrevistas a políticos en TV (Irritación cognitiva).
2. Incluso estando en casa pienso a menudo en los problemas políticos nacionales (Irritación cognitiva).
3. Me pongo de mal humor cuando otros se me acercan (Irritación emocional).
4. Incluso en mis vacaciones pienso en los problemas políticos de mi país o de mi ciudad (Irritación cognitiva).
5. De vez en cuando me siento como un atado de nervios (Irritación emocional).
6. Me enojo rápidamente, especialmente cuando escucho hablar a políticos (Irritación emocional).
7. Me irrito fácilmente aunque no quiero que esto suceda. (Irritación emocional).
8. Cuando llego a casa cansado, después del trabajo, me siento bastante irritable (Irritación emocional).

#### 2.2.1. Happiness

To measure happiness, the Happiness Scale for Adults (EFPA in its Spanish acronym) was used (Moyano et al., 2018). The EFPA was initially developed and validated with Chilean adult participants and consists of 21 five-point Likert-type items (1 = totally disagree to 5 = totally agree) that measure overall happiness and three happiness-relevant dimensions: Psychological State (e.g., “I do not feel happy most of the time.” Reverse item), Family (e.g., “I have the support of my family”), and Accomplishment-Optimism (e.g., “I have met the objectives that I have set for myself”). In the current study, as our predictions point to effects on overall happiness, only scores on the total scales were used. The EFPA has shown adequate reliability in the past (Moyano-Díaz, et al., 2021), and, in the current study, its reliability was also adequate (*α* = 0.92).

#### 2.2.2. Political beliefs

To measure participants’ political interest, electoral participation, and political hopelessness, three single seven-point Likert-type items were used (1 = totally disagree to 7 = totally agree). Following the European Social Survey (2006), political interest was measured by asking participants to indicate: “To what extent would you say that you are interested in politics?” as a single value item using the Likert-type response format from 1 (not at all) to 5 (a lot). Similarly, electoral participation was measured by asking participants to indicate to what extent they agree with the statement “I always vote in elections” with a single seven-point Likert-type item were used (1 = totally disagree to 7 = totally agree). Finally, to evaluate participants’ belief of political hopelessness concerning delinquency, they were asked to indicate to what extent they agree with the statement “Left- and right-wing democratic governments are helpless when it comes to fighting delinquency” with a single seven-point Likert-type item were used (1 = totally disagree to 7 = totally agree).

### 2.3. Procedure

The participants were contacted through social networks and emails between January and April 2022, inviting them to answer an online survey with questions about what Chile is like today. A link (http) was sent to respond to the survey and those who agreed to participate did so through informed consent. They then proceeded to complete the instruments related to anomie, irritation, and happiness and a series of questions associated with their political beliefs (interest, voting and helplessness). Finally, they were asked to provide demographic information (age, sex) and were thanked for their participation.

## 3. Results

### 3.1. Presence of anomie and its relationship to irritation and happiness

Following Teymoori et al. (2017), Figure 1 shows that, in our sample, people experience great anomie, represented by relatively high perceptions of the breakdown of leadership and of the social fabric.

**Figure 1:**
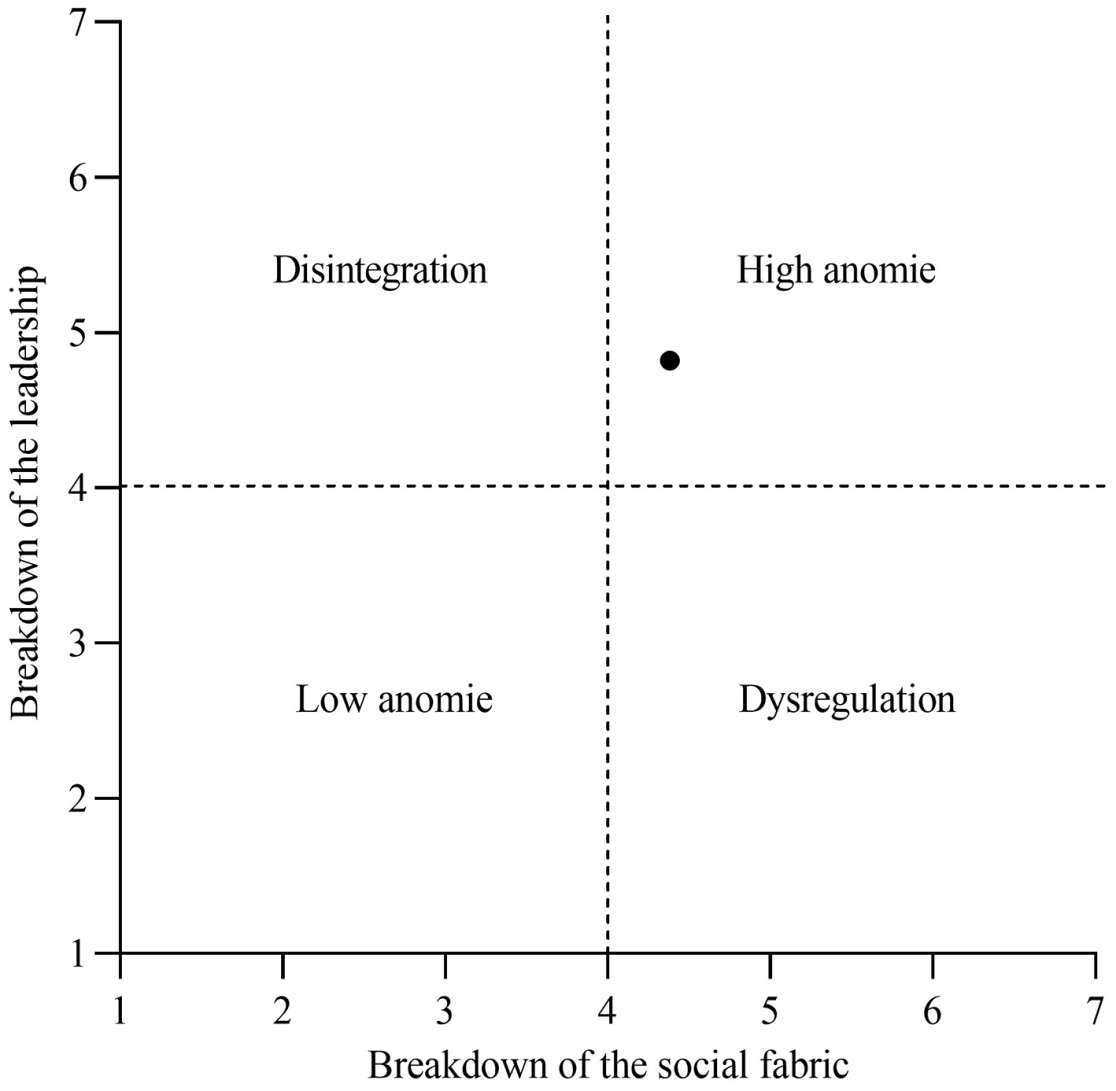
State of anomie based on means for rupture of the social fabric (4.38) and rupture of leadership (4.82) where the black dot indicates the location of Chile. Adapted from “Towards a psychological analysis of anomie” by Teymoori et al. (2017), *Political Psychology*, 38:1014 (doi: 10.1111/pops.12377).

Additionally, we expected to find anomie was positively related to irritation and negatively related to happiness, with both having been verified. The means and standard deviations, as well as correlations, between anomie, its core dimensions, irritation, and happiness are presented in Table 1. Both anomie and its core dimensions—breakdown of the social fabric and of leadership—were positively associated with irritation and negatively associated with happiness. Furthermore, irritation showed a moderate negative association with happiness.

**Table 1:** Descriptive statistics and inter-correlations between anomie and its core dimensions, irritation, and happiness.

	1	2	3	4	5
1. Anomie	--	0.77**	0.82**	0.21**	– 0.17*
2. Breakdown of social fabric		--	0.27**	0.17*	– 0.17*
3. Breakdown of leadership			--	0.16*	– 0.10
4. Irritation				--	−0.47**
5. Happiness					--
*M*	4.60	4.38	4.82	3.78	3.91
SD	0.77	0.92	1.01	1.19	0.69

*p < 0.05, **p < 0.01

**Figure 2:**
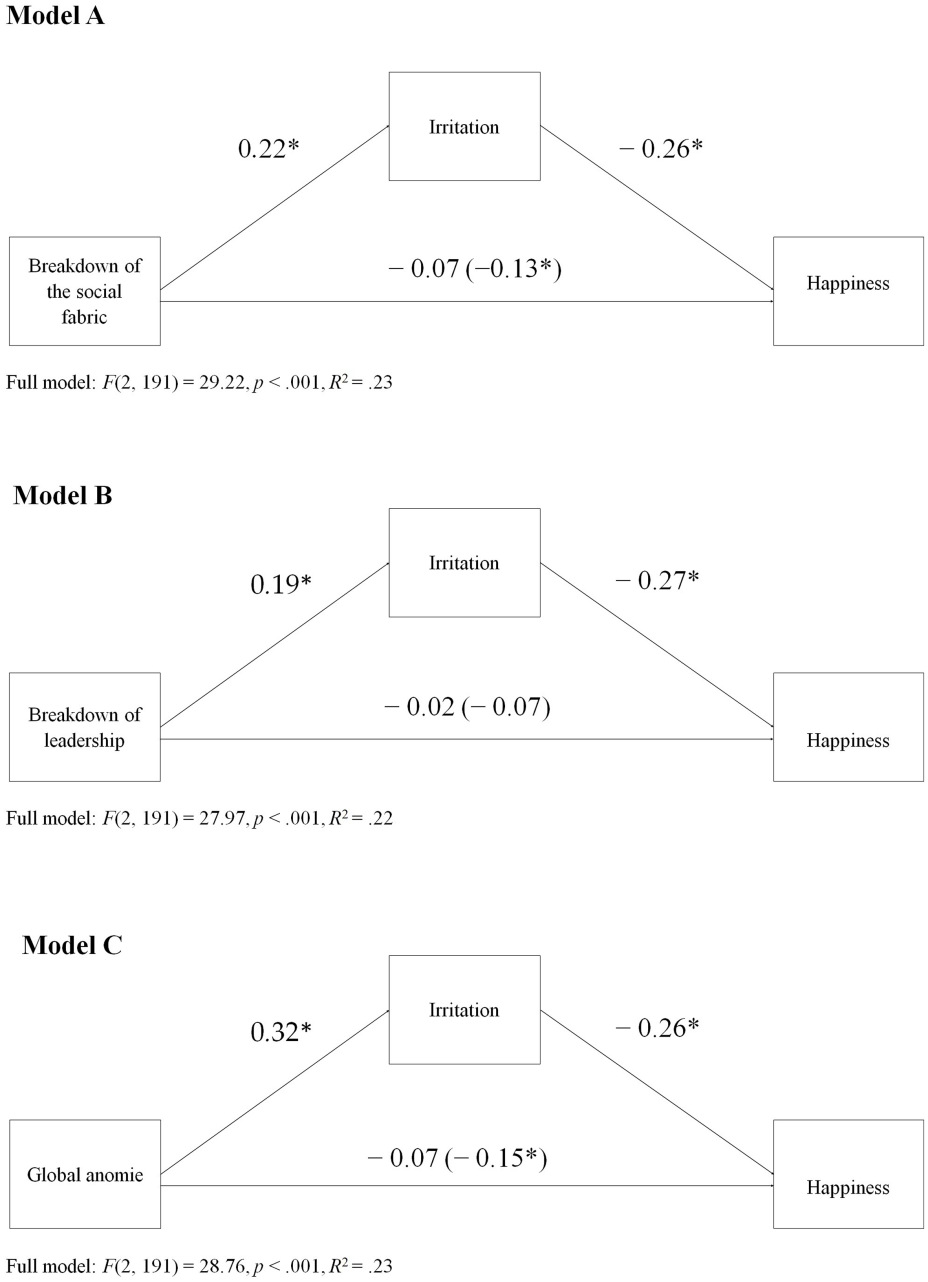
Influence of anomie and its core dimensions on happiness through irritation.

To evaluate whether the associations between happiness and the breakdown of the social fabric and of leadership are explained by an increase in irritation, two mediation analyzes were conducted (Hayes, 2012). As can be seen in Figure 2 (Model A), the breakdown of the social fabric positively influenced irritation, *b* = 0.22, SE = 0.09, *p* = 0.016, 95% CI [0.04, 0.40], and irritation, in turn, was negatively related to happiness, *b* = − 0.26, SE = 0.03, *p* < 0.001, 95% CI [− 0.33, − 0.19]. Importantly, the results showed a negative indirect effect of the breakdown of the social fabric on happiness through irritation, *b* = − 0.05, SE = 0.02, 95% CI [− 0.10, − 0.01]. In other words, the influence of the breakdown of the social fabric on happiness was explained by its negative association with irritation.

Similarly, as can be seen in Figure 2 (Model B), the results showed that the breakdown of leadership also positively influenced irritation, *b* = 0.19, SE = 0.08, *p* = 0.021, 95% CI [0.30, 0.35], and this, in turn, negatively affected happiness, *b* = − 0.27, *SE* = 0.03, *p* < 0.001, 95% CI [− 0.34, − 0.19]. Although the results showed neither a direct effect, *b* = − 0.02 *SE* = 0.04, *p* = 0.62, 95% CI [− 0.10, 0.06] nor a total effect, *b* = − 0.07, *SE* = 0.04, *p* = 0.12, 95% CI [− 0.17, 0.02], of the breakdown of leadership on happiness, they did reveal a negative indirect effect through irritation, *b* = − 0.05, *SE* = 0.02, 95% CI [− 0.10, − 0.01].

Finally, regarding the overall measure of anomie, and as seen in Figure 2 (Model C), while irritation was positively influenced by anomie, *b* = 0.32, *SE* = 0.10, *p* = 0.003, 95% CI [0.11, 0.53], irritation itself was negatively related to happiness, *b* = − 0.26, *SE* = 0.03, *p* = 0.62, 95% CI [− 0.34, − 0.19]. Importantly, in a similar way to our findings for the effects of the breakdown of the social fabric, the results showed a negative indirect effect of global anomie on happiness through irritation, *b* = − 0.08, SE = 0.03, 95% CI [− 0.15, − 0.03].

### 3.2. Associations between anomie and political interest, electoral participation, and political hopelessness concerning delinquency

Correlation analyzes were conducted to test how anomie and its core dimensions relate to indicators of political interest, electoral participation, and political hopelessness. While the overall measure of anomie was not related to any political belief, *r*_interest_ = − 0.10, *p* = 0.158; *r*_participation_ = − 0.08, *p* = 0.236; *r*_hopeless_ = 0.10, *p* = 0.151, both of its core dimensions—breakdown of the social fabric and breakdown of leadership—showed significant associations with political hopelessness and political interest, respectively. In particular, the breakdown of the social fabric was positively related to a belief that left and right-wing democratic governments are helpless when it comes to fighting delinquency, *r* = 0.15, *p* = 0.027. The breakdown of leadership, on the other hand, was negatively related to political interest, *r* = − 0.18, *p* = 0.010. No other significant associations were observed.

## 4. Discussion

The Chilean social outbreak of 18 October 2019 mobilized collective action across the country. Although the tensions decreased with the agreement for a new political constitution (which was later electorally rejected by the citizens) and the advent of the COVID-19 pandemic, we argue that there is a long-standing anomic process of which the social outbreak is part, and at the same time, dialectically, it feeds back into anomie (Moyano-Díaz et al., 2021), which still persists today, negatively affecting people’s well-being through greater irritation.

This study has some methodological limitations that encourage us to interpret its results with caution. For example, the type of sampling, which is not representative of the Chilean population; and its correlational design, which does not allow a comparison of longitudinal changes in the participants. However, with the purpose of highlighting the theoretical scope of the concepts treated according to the data obtained, we will develop some conclusions based on the analytical richness offered by the concept of anomie and its relationship with irritation and well-being.

### 4.1. An anomic process in Chile

People perceive both the social fabric and leadership to be fractured. Descriptively, our results show that Chile is located in the quadrant of high anomie. The average perception of a breakdown of leadership was higher than that of a breakdown in the social fabric (4.82 vs.4.38). Provocative and/or sarcastic statements made by right-wing government ministers, such as making improper jokes regarding the increase in fares in public transport (for example, the Minister of Transport during the last right-wing government said that people should “get up earlier” to use cheaper tickets), or regarding the few products that were actually cheaper during that time (Minister of Finance of the right-wing government, “buy flowers”) or suggesting that improving the public infrastructure of schools is the responsibility of the users and not the state (for example, the right-wing Minister of Education said “why do not they run a bingo? Why do I have to leave Santiago to fix the roof of a gym?”), could have contributed to this difference. Our results also showed that, while the breakdown of both the social fabric and of leadership were negatively related to happiness, the negative relationship between the former and happiness was stronger than that of the latter and happiness. Apparently, then, there is a greater negative effect on citizens’ happiness when they perceive a breakdown in the social fabric rather than in leadership. Possible explanations for this may lie in a growing distrust in State law enforcement agencies, such as the police, and the qualitative change in robberies, in which, over the last 2 years, criminals have shown a greater disregard for life, with insecurity and social distrust on the increase. Additionally, as regards leadership, Chilean politics, in recent years, has taken on a tone that is sometimes “showbiz” and sometimes sarcastic. Political leaders are frequently seen on various television entertainment shows and are not taken seriously by viewers. This is aligned with national surveys in which politicians and political parties often receive the lowest ratings of trust and respectability from citizens (24 Horas, 2022). Thus, while, in 1990 and 2000, 22% of citizens agreed that most people could be trusted; this fell to only 14% in 2018. Regarding whether Congress could be trusted somewhat or a lot, in 1990, 63% agreed with this, but, in 2018, this dropped to just 18%, with political parties down from 28 to 16%, the government down from 50 to 37%, and the judiciary falling from 45 to 30% (World Values Survey, Séptima Ola, 2018).

Furthermore, following Teymoori et al. (2017), the breakdown of leadership may have facilitated the weakening of the social fabric. Leaders that are perceived as ineffective and unfair constitute a threat to social cohesion, with nepotism and despotism particularly leading to marginalization, eroding the sense of belonging to a community, trust in others, and the perception of agreed moral standards. Additionally, viewers, and citizens in general, may distance themselves from politicians who indulge in disputes on daily media shows over issues that have little or nothing to do with the interests of the people, and then do not allow their well-being or happiness to be affected by this. In addition, it is an enormously vicarious experience, since, in reality, ordinary people have very few possibilities of interaction with political leaders.

### 4.2. Anomie, irritation, and well-being

Our results provided partial evidence in favor of our hypothesis. As mentioned, both anomie and its core dimensions—the breakdown of the social fabric and breakdown of leadership—were negatively related to happiness. These results are consistent with those of Laca Arocena et al. (2011), who found that anomie was negatively associated with social well-being, and with those reported by Teymoori et al. (2016). Furthermore, anomie and its core dimensions were positively associated with irritation, providing preliminary evidence of its role as a possible mediator. However, while irritation explained the association between global anomie and happiness, and between the breakdown of the social fabric and happiness, the results on the relationship between the breakdown of leadership and happiness were less clear. Specifically, while the results did show an indirect effect of irritation on the relationship between the breakdown of leadership and happiness, they showed neither a total nor a direct effect. One possible explanation is other (unmeasured) constructs might be at play. Indeed, this indirect-only effect (Zhao et al., 2010) might occur in the presence of a competing mediator that has yet to be identified. This competing mechanism would likely be positively influenced by anomie but positively related to happiness, and would thus cancel out a potential direct effect of anomie on the dependent variable (e.g., inconsistent mediation; MacKinnon et al., 2000). More research is needed to explore this alternative.

### 4.3. Anomie and political beliefs

Our results showed that perceptions of anomie relate to political beliefs. In particular, the more people perceive a breakdown of the social fabric, the more they come to believe that governments are helpless to fight against delinquency. Additionally, within Latin America, Chile has historically been a country with low crime rates, and in recent years, as mentioned, the emergence of gangs and organized crime have driven its crime rate to unprecedented levels. Underlying this may be what Dolliver (2015) describes as anomic cultural pressures to succeed interacting with an economically dominated institutional balance of power, with the result likely being high crime rates. In the same way, the greater people’s perception of a breakdown in leadership, the less political interest they report, which coincides with the findings of Laca Arocena et al. (2011).

In this study, we observed that anomie and irritation are negatively and significantly correlated with happiness, although irritation has a stronger negative correlation with happiness than anomie. Thus, it is of interest to determine the specific aspects of breakdown in leadership and in the social fabric that most irritate citizens. It would be useful to verify, for example, what generates more irritation, sarcasm by authorities or government leaders, or the incompetence and procrastination of parliamentarians in the formulation of laws relevant to life in society. Bills on crime, social security and pension system, immigration, and the environment remain dormant for long periods in Congress. More recently, the excessive time taken to reach an agreement on the mechanism to formulate a new constitutional project, or the inappropriate use of money in parliamentary functions, are new and likely highly significant sources of citizen irritation. Could factors such as those mentioned herein eventually help predict new social outbreaks? It is necessary to measure these specific aspects to identify their relative weight in the irritation of citizens.

### 4.4. Limitations and future developments

First, although our sample of adults enriches the analysis with respect to previous Chilean studies carried out only with students, as we mentioned it is limited in terms of representativeness of the Chilean population. Future developments should build on our results and explore the mechanisms linking anomie and happiness in a larger and more diverse national sample. Second, while here it is hypothesized and verified that anomie impacts happiness through increased irritation, as we also mentioned the correlational design used does not allow causal conclusions to be drawn. Future studies could get around this problem by applying longitudinal designs and performing natural experiments that allow researchers to test whether changing levels of anomie before and after major political and social cycles lead to varying levels of happiness. Third, from a conceptual and explanatory point of view, the anomie expressed in a significant increase in crime and in social outbreaks with street violence consisting of the destruction of street furniture, burning of busses, churches, and commercial establishments generates fear, and its contribution as a mediating mechanism—in addition to irritation—on the decrease in happiness could be evaluated in future studies.

Finally, although we report the reliability of the measures used here, the current study was not intended as a psychometric exploration of the anomie and irritation scales in a Chilean sample, and therefore a deeper exploration of the psychometric properties of these measures is required.

## Data availability statement

The datasets presented in this article are not readily available because the datasets include the private information of participants. The datasets will be available upon request to researchers who are members of official institutions, after contact and identification with the corresponding author.

## Ethics statement

The studies involving human participants were reviewed and approved by Comité de Ética de la Facultad de Psicología de Universidad de Talca. The patients/participants provided their written informed consent to participate in this study.

## Author contributions

EM-D designed the research and data collection, and wrote the article. GP-V analyzed the data and contributed to writing the article. PO-J contributed to the analysis and interpretation of data and to writing the paper, and YM-L contributed to writing the article and preparing the manuscript format for submission and corrections.

## Conflict of interest

The authors declare that the research was conducted in the absence of any commercial or financial relationships that could be construed as a potential conflict of interest.

## Publisher’s note

All claims expressed in this article are solely those of the authors and do not necessarily represent those of their affiliated organizations, or those of the publisher, the editors and the reviewers. Any product that may be evaluated in this article, or claim that may be made by its manufacturer, is not guaranteed or endorsed by the publisher.
